# When it all falls down: the relationship between intuitive physics and spatial cognition

**DOI:** 10.1186/s41235-020-00224-7

**Published:** 2020-05-19

**Authors:** Alex Mitko, Jason Fischer

**Affiliations:** Department of Psychological and Brain Sciences, Johns Hopkins University, 3400 North Charles Street, Baltimore, MD 21218 USA

**Keywords:** Intuitive physics, Individual differences, Mental rotation, Paper folding, Working memory

## Abstract

Our intuitive understanding of physical dynamics is crucial in daily life. When we fill a coffee cup, stack items in a refrigerator, or navigate around a slippery patch of ice, we draw on our intuitions about how physical interactions will unfold. What mental machinery underlies our ability to form such inferences? Numerous aspects of cognition must contribute - for example, spatial thinking, temporal prediction, and working memory, to name a few. Is intuitive physics merely the sum of its parts - a collection of these and other related abilities that we apply to physical scenarios as we would to other tasks? Or does physical reasoning rest on something extra - a devoted set of mental resources that takes information from other cognitive systems as inputs? Here, we take a key step in addressing this question by relating individual differences on a physical prediction task to performance on spatial tasks, which may be most likely to account for intuitive physics abilities given the fundamentally spatial nature of physical interactions. To what degree can physical prediction performance be disentangled from spatial thinking? We tested 100 online participants in an “Unstable Towers” task and measures of spatial cognition and working memory. We found a positive relationship between intuitive physics and spatial skills, but there were substantial, reliable individual differences in physical prediction ability that could not be accounted for by spatial measures or working memory. Our findings point toward the separability of intuitive physics from spatial cognition.

## Significance statement

To effectively interact with the world, we must draw on our intuitions about how objects will behave when we act on them and when they interact with each other. This mental framework for understanding and predicting physical dynamics - termed *intuitive physics* - allows us to fluidly engage with a world where things roll, swing, bounce, balance, slosh, slide, and collide. Despite the critical importance of our intuitive physics abilities, their underlying mental processes are not well-understood. Our work here tests the relationship between intuitive physics and spatial cognition. To what degree are the mental resources for understanding and predicting physical dynamics shared with those that allow us to apprehend the geometry of a place and 3D structure of objects? Here, we found a positive relationship between intuitive physics and spatial cognition in our tasks, but the two were separable - variation in peoples’ physical prediction abilities could not be fully explained by their spatial skills. Our results point toward the possibility that we possess some specialized mental resources for understanding and predicting physical behavior - a physics engine in the mind. Better understanding this mental physics engine will help us address the needs of those who have difficulty with physical prediction (for example, those with apraxia or Williams syndrome), help us build machines that can more fluidly interact with their environments and with humans, and further our basic understanding of the architecture of the mind.

## Introduction

Our ability to interpret and predict the physical dynamics of a scene is crucial in everyday life. To pick up a coffee cup with the correct force, stack a pile of dishes so that it will not fall, or grab a rebound after it bounces off of the rim, we must anticipate how objects will behave when they interact with each other and with our own bodies. Our experience performing such tasks is not one of crunching the equations of Newtonian dynamics. Rather, we have an intuitive sense of how objects will behave - we “see” that the dishes are unstable or that the basketball will bounce to the right. While a body of early work revealed some systematic errors in humans’ physical judgements (Caramazza, McCloskey, & Green, 1981; Gilden & Proffitt, 1989; Kaiser, Jonides, & Alexander, 1986; McCloskey, 1983), more recent work has focused on our competencies - humans can often form accurate and nuanced physical predictions under the kinds of conditions that we encounter in daily life (Battaglia, Hamrick, & Tenenbaum, 2013; Sanborn, Mansinghka, & Griffiths, 2013).

What mental resources do we rely on to predict the physical dynamics of everyday scenes? One possibility is that we draw in an ad hoc fashion on a collection of related abilities such as spatial reasoning, working memory, and temporal prediction. Physical inference necessarily involves these aspects of cognition because physical interactions play out over space and time, often involving multiple objects interacting on fast timescales. For example, predicting how two objects will bounce when they collide requires tracking their positions and orientations over time and determining how the surfaces of their 3-dimensional structure will come into contact. It is possible that our physical intuitions arise purely from a collection of other mental systems working in concert - physical prediction may be just another kind of everyday challenge that we solve with the broad set of cognitive abilities at our disposal.

An alternative possibility is that our physical inferences rely on a specialized set of mental resources that are devoted to interpreting and predicting the physical interactions in a scene - a physics engine in the mind (Battaglia et al., 2013; Fischer, 2020; Kubricht, Holyoak, & Lu, 2017; Ullman, Spelke, Battaglia, & Tenenbaum, 2017). In this view, other facets of cognition such as spatial reasoning and working memory would serve as inputs to a mental physics engine that makes use of such information to predict physical dynamics under Newtonian principles. Under this proposal, while many inputs from other cognitive systems make indispensable contributions to our physical intuitions, they are not the whole story. How separable is intuitive physics from these other cognitive domains? To date, there has been little work to test the relationship between physical cognition and other closely related abilities. Studies of individuals with autism and Williams syndrome have shown that intuitive physics is dissociable from social cognition (Baron-Cohen, Wheelwright, Spong, Scahill, & Lawson, 2001; Kamps et al., 2017), which relies on its own domain-specific system (Saxe & Baron-Cohen, 2006), but studies of the relationship between intuitive physics and more closely related domains such as spatial thinking and working memory are lacking.

Here, we test the relationship between physical prediction abilities and performance on measures of spatial cognition and working memory using an individual differences approach. We focus on spatial cognition because (1) intuitive physics is fundamentally spatial, with our physical prediction abilities hinging on the need to track the 3-D structure and orientations of objects as they move and rotate, (2) physical prediction and spatial manipulation engage highly similar sets of brain regions (Fischer, Mikhael, Tenenbaum, & Kanwisher, 2016; Richter et al., 2000; Vingerhoets, De Lange, Vandemaele, Deblaere, & Achten, 2002), and (3) joint impairments in intuitive physics and spatial cognition have been observed in a clinical population (Kamps et al., 2017). We aimed to characterize individual differences in physical prediction abilities and test whether they could be accounted for by individual differences in spatial reasoning, while testing working memory as a comparison condition. Our reasoning is that if the mental resources that support physical prediction are at least somewhat independent of these other abilities, then people will vary in their physical prediction abilities in a way that cannot be fully accounted for by the other two.

To capture individual differences in physical prediction abilities, we refined a task that has recently been employed in a host of studies, the Unstable Towers task (Battaglia et al., 2013; Fischer et al., 2016; Hamrick, Battaglia, Griffiths, & Tenenbaum, 2016). In this task, participants see a tower of blocks that is about to topple and must decide how it will fall, reporting aspects of the outcome such as the direction or spread of the final resting state of the blocks. It is important to note that the Unstable Towers task captures only one facet of the vast and varied space of physical inferences that we make in everyday life. A host of other tasks have recently been used to explore other aspects of intuitive physics, such as predicting the trajectories of projectiles and bouncing balls (K. A. Smith, Battaglia, & Vul, 2018; K. A. Smith & Vul, 2013, 2013), inferring the relative masses of objects from observations of collisions (Flynn, 1994; Gilden & Proffitt, 1994; Sanborn et al., 2013), predicting the behaviors of liquids (Bates, Battaglia, Yildirim, & Tenenbaum, 2015; Schwartz & Black, 1999), judging causality through counterfactual reasoning (Gerstenberg, Goodman, Lagnado, & Tenenbaum, 2015; Gerstenberg, Peterson, Goodman, Lagnado, & Tenenbaum, 2017), and many others. The demands of the unstable towers task and the mechanisms for solving it may differ from other intuitive physics tasks (see “Discussion”), but here we have chosen to use it as a starting point for our investigation of the relationship between intuitive physics and other aspects of cognition both because stability judgements are common and crucial in daily life and because the task has factored prominently into recent work on the mental and neural mechanisms of intuitive physics. Still, we acknowledge that this study is just the beginning of a program of work that will be required to understand how intuitive physics relates to other cognitive domains.

A central challenge in the endeavor of comparing physical prediction abilities with other cognitive abilities across individuals was establishing a task that could reliably capture individual differences in physical prediction abilities. While spatial cognition and working memory tasks are in wide use and have been refined to measure individual differences (Conway, Kane, & Engle, 2003; Hegarty & Waller, 2005; Just & Carpenter, 1992; Yılmaz, 2017) and train transferrable skills (Uttal et al., 2013; Wright, Thompson, Ganis, Newcombe, & Kosslyn, 2008), no such measures yet exist to capture individual differences in physical prediction abilities in adults. One test with physical diagrams has been described in the literature on autism spectrum disorders to characterize variations in physical problem solving (Baron-Cohen et al., 2001), but the problems on this test may tap into different abilities than we are interested in here. People can display striking misconceptions in such physical diagramming tasks (Caramazza et al., 1981; McCloskey, Caramazza, & Green, 1980), even when their physical predictions in more naturalistic tasks that mirror the kinds of scenarios they encounter in everyday life are accurate and nuanced (Flynn, 1994; Kaiser et al., 1986; K. Smith, Battaglia, & Vul, 2013). Performance of physical diagramming tasks highlights a critical puzzle about the nature of our physical cognition, but may not necessarily capture the breadth of our physical judgement capabilities under everyday circumstances. Expanding literature on this topic has shown that people can make accurate and detailed physical predictions when tested with tasks that more closely reflect the automatic, implicit physical judgements that people make in daily life (Battaglia et al., 2013; Ullman et al., 2017). Thus, our work in this study began with the task of adapting a more naturalistic task (the unstable towers task) to reliably capture individual differences in physical prediction abilities. We then leveraged this measure of individual differences to examine the relationship between performance on the towers task and other tasks that measure spatial reasoning and working memory. By determining how much of the variation in our towers task was captured by the other tasks, we established an initial assessment of the separability of physical prediction abilities from these other domains.

## Methods

### Participants

All participants were recruited on Amazon’s Mechanical Turk. Participants were required to be 18–35 years of age and complete the study from within the USA. For this study, we chose to limit our participant pool to younger adults because the effects of aging on intuitive physics abilities are not well-characterized. For other tasks that we consider here, such as mental rotation, there are documented effects of aging (Berg, Hertzog, & Hunt, 1982). Because our key questions here did not specifically concern effects of age, we chose to limit this potential source of variability by restricting the age range of our participants. The order of the five tasks was randomized for each participant, and participants were required to complete one task at a time. Based on piloting of the unstable towers task, we determined that 100 participants would be sufficient to provide power of 0.95 with an alpha of .001 in our correlation analyses (we used a conservative alpha level to account for the fact that we planned to compute multiple tests of correlation and correct for multiple comparisons). In total, 129 Mechanical Turk workers completed the tasks, but 27 of them were excluded before the analyses for the following reasons: 17 participants did not demonstrate an understanding of one or more of the tasks (determined by blank responses for an entire task or through comments left at the end of the task that reflected a misunderstanding), 5 participants had malfunctions with the data-saving file, 2 participants were not in the USA, and 3 participants attempted to complete multiple tasks at the same time. Two participants were excluded from analysis for falling below 5 standard deviations on the working memory tasks’ symmetry judgements and lexical decisions (these secondary tasks were not used for the main analysis, but provided a means of assessing whether participants faithfully performed the working memory tasks). This left a total of 100 participants for analysis. Participants were paid US$8 to complete the study and provided anonymous informed consent in accordance with Johns Hopkins University Institutional Review Board (IRB) protocols.

### Unstable towers task

#### Design

**Fig. 1:**
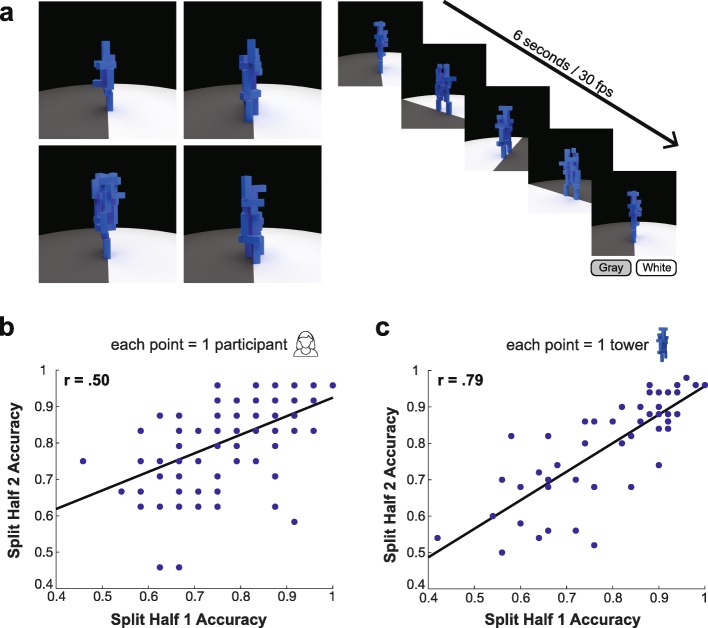
Reliable individual differences in the Unstable Towers task. **a** Left, examples of unstable tower stimuli used in the task. Right, each tower was displayed in a 6 s video at 30 frames per second that circled the tower in 360°. After viewing each video, participants reported which side of the platform the majority of blocks would land on - gray or white. **b** Performance on the towers task was reliable across independent sets of stimuli. We split the set of towers into two halves (see “Methods”) and computed performance in the two stimulus sets (each plotted point is one participant). Performance across the split halves was significantly correlated (*r* (98) = 0.50, *p* < .001) and spanned the range from near chance to near perfect, indicating that our version of the towers task is a reliable and sensitive measure of individual differences. **c** The difficulty of assessing each tower was reliable across independent sets of observers. We split the participants into two groups and computed performance for each tower stimulus within each group (plotted points are individual towers). Accuracy was highly significantly correlated across split halves (*r* (98) = .79, *p* < .001) and spanned a broad range of difficulty

We developed a modified version of the unstable towers task (Battaglia et al., 2013; Fischer et al., 2016; Hamrick et al., 2016) in which subjects must predict how an unstable tower of blocks will tumble. On every trial, participants viewed an unstable tower in a 360° panoramic video that lasted for 6 s at 30 frames per second (Fig. 1a). The towers were centered on a platform that was split in the middle by color (either gray or white). Participants had to judge on which side the majority of the blocks would come to rest after the tower had fallen. Participants first saw a practice trial that showed a full video of a tower falling so that they could develop a sense of the materials and mass used for the blocks. They then viewed 48 tower videos and made judgements about how the blocks would fall without receiving feedback. After viewing each video, participants made their responses by clicking buttons under the two sides of the platform labeled “gray” or “white”. Participants were required to make a response in order to advance to the next trial. We varied the number of blocks used to construct the towers: there could be 11, 13, 15, 17, 19, or 21 blocks within a tower; we constructed 8 towers with each number of blocks, with 4 falling to the gray side and 4 falling to the white side. We presented the towers in a random order to each participant. The last frame of each video remained on the screen until the participant advanced to the next trial.

#### Stimuli

All towers were constructed and rendered using Blender 3-D modeling software (http://www.blender.org), and physical outcomes were assessed using simulations run in Blender’s built-in Bullet physics engine. It is important to note that these non-probabilistic simulations do not always capture human judgments well (Battaglia et al., 2013). There are cases where human judgments differ from the results of a single deterministic simulation in ways that probabilistic models would also differ from non-probabilistic ones. It would not make sense to label observers’ judgements in these cases as errors when they in fact reflect reasonable assessments under uncertainty, and here we took steps to avoid such cases. During piloting, we identified towers for which human observers agreed with the Blender simulation outcomes the majority of the time (> 55%). We limited our final stimulus set to those towers where there was general agreement between pilot observers and simulation outcomes. Ground truth in this case thus refers to an outcome that both the Blender simulation and observers’ intuitions generally agreed on. In the main experiment, to evaluate participants’ prediction accuracy, we compared their responses with these outcomes that our piloting established to be generally consistent between the simulation outcomes and pilot subjects’ judgments.

We used Blender to create the towers stimuli for several important reasons. First, it allowed us to construct towers that were unstable and ready to fall, but did not require any supporting structure to be part of the visual scene (taking a snapshot of an unstable tower in the real world would be challenging). Second, Blender allowed perfect control over variables such as scene lighting, gravity, object mass, and the friction of surfaces. Third, Blender allowed for rapid prototyping and reconfiguration of the arrangement of blocks in the scene. Producing a set of scenes that were challenging but possible to predict required several iterations of reconfiguring towers and piloting to remove those that were too easy or too difficult. All data shown in this study are independent of the data used for piloting, collected after the final set of towers had been established.

### Spatial tasks

#### Paper Folding Test

**Fig. 2:**
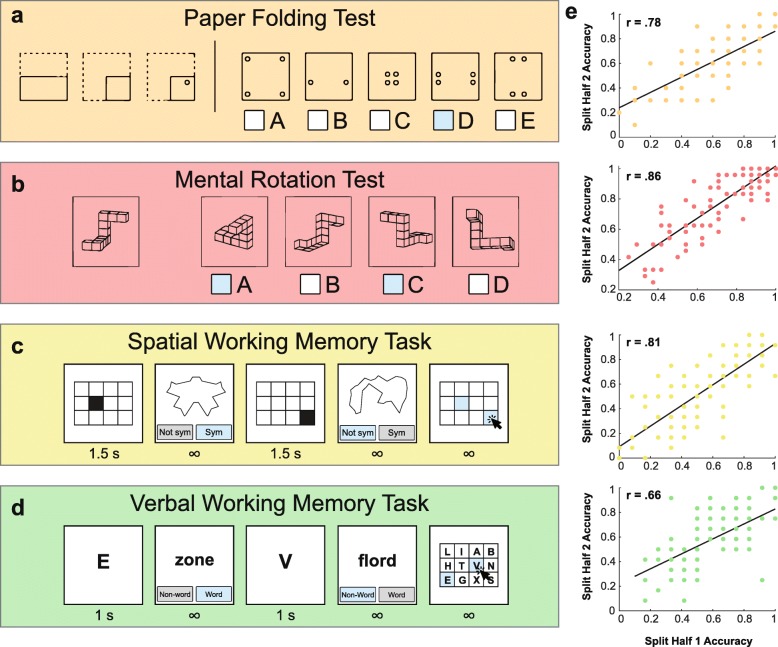
Spatial reasoning and working memory tests. **a** Example item from the Paper Folding Test (Ekstrom, Dermen, & Harman, 1976). The three items on the left indicate how a square piece of paper is folded and hole-punched. Participants must determine where the holes would be located if the paper was unfolded back to its original form. **b** Example question from the Mental Rotation Test (Peters et al., 1995; Shepard & Metzler, 1971; Vandenberg & Kuse, 1978). Participants must determine which two items on the right are rotated versions of the item on the left. The remaining two incorrect choices are structurally different to the target item. **c** General layout of the spatial working memory task. Participants were required to remember a series of filled in grid positions while performing a secondary symmetry judgement (see “Methods”). The symmetry judgement required participants to determine whether the shape was perfectly symmetrical when split vertically down the middle. The number of grid positions that participants were required to hold in memory varied between three and eight on different trials. At the end of each trial, participants reported the remembered grid positions by clicking within a blank grid (far right panel). **d** General layout of the verbal working memory task. Participants were required to remember a series of letters while performing a secondary lexical decision task (see “Methods”). In the lexical decision task, participants reported whether a word was a real or fake English word. The number of letters that participants were required to hold in memory varied between three and eight on different trials. At the end of each trial, participants reported the remembered series of letters by clicking within a grid (far right panel). **e** Split-half reliability for each task. The trials from each task were split into two sets and performance was computed for each independent half (each data point represents the performance of one participant). All split-half correlations were statistically significant (*p* < .001)

We used the Paper Folding Test developed by Ekstrom, Dermen, and Harman, (1976) as a test of spatial mental manipulation. In this task, participants were asked to imagine a square piece of paper being folded and hole-punched through all of the folded layers (Fig. 2a). For each question, the first series of images depicted how a square piece of paper was folded and then a hole was punched. A second series of images showed possible locations of the holes when the paper was unfolded back to its original state. An example of a correctly answered question was shown in the task instructions before starting. There were 20 questions in total, all available to participants at the same time in order to most closely match the conditions of the original pencil and paper version of the test. Participants were required to select one of the five options for each question. There was no time limit for completing the task. Accuracy was determined by dividing the total number of correct choices by the total number of questions.

#### Mental Rotations Test

As a second measure of spatial mental manipulation abilities, we used the Mental Rotations Test (Peters et al., 1995; Shepard & Metzler, 1971; Vandenberg & Kuse, 1978). The test consisted of 24 questions in which a target stimulus of connected blocks was shown, along with four options reflecting possible rotated states of the same block object (Fig. 2b). Two of the options were rotated versions of the target stimulus, whereas the other two other options depicted structurally different objects. Participants were instructed to select the two rotated versions of each target object. Examples of correctly answered questions were shown in the task instructions before starting. All 24 questions were shown on the screen at the same time in accordance with the original pencil and paper test, and participants were required to select two options for each question. There was no time limit for completing the task. Accuracy was determined by dividing the total number of correct choices by 48, as there were two correct answers for each question. Note that our scoring scheme differs from how the test is sometimes scored by awarding 1 point for each fully correct item. Our approach provided a more continuous measure of performance for the sake of testing correlation with other tasks - selecting one correct option still earned the participant partial credit.

### Working memory tasks

#### Spatial working memory

Our test of spatial working memory was based on commonly used complex span tasks (e.g., Blacker, Negoita, Ewen, & Courtney, 2017; Chein & Morrison, 2010). We required participants to perform two tasks in alternation (Fig. 2c). First, they saw a 3 × 4 grid with a single black square filled in for 1500 ms. Participants were instructed to remember the position of this black square as they would be asked to recall it later. After a 750 ms delay, participants were then presented with a shape and asked to determine whether it was perfectly symmetrical if it were split vertically down the middle, providing a response by clicking one of two buttons labeled “Symmetrical” and “NOT Symmetrical”. This process was repeated a total of 3–8 times, and at the end of the stimulus sequence, a black response grid appeared. Participants used their mouse to select the positions of the filled-in squares that they remembered from the sequence. Selected grid locations were highlighted in yellow and participants were able to select and unselect positions as many times as they wanted. When they were finished selecting positions in the grid, participants pressed a “Submit” button and started a new sequence of remembered locations and symmetry judgements. Participants completed four trials of each sequence length. Order of the position selection was not emphasized nor analyzed. The grid locations were randomly generated for each participant. All of the shapes were generated in Matlab prior to conducting the study. Participants saw a total of 132 shapes in a randomized order, half symmetrical and half non-symmetrical. Responses on the grid position memory task were only considered correct if all of the correct grid positions were selected and no incorrect positions were selected.

#### Verbal working memory

Our verbal working memory task was similar in design to the spatial working memory task, and based on commonly used verbal complex span tasks (e.g., Chein & Morrison, 2010). At the start of the task, participants were presented with a series of capital letters in the middle of the screen for 1000 ms each, and asked to remember the full sequence of letters (Fig. 2d). After seeing each letter, participants performed a secondary task: they saw either a word or non-word and had to determine whether the presented item was a word recognized by the English language dictionary, or a non-word generated using the English Lexicon Project generator (Balota et al., 2007). They indicated their choice by clicking a button labeled “NON-WORD” or “WORD”. After 3–8 capital letters were shown, the response page was shown, which consisted of a 3 × 4 grid filled with capital letters that included the target remembered letters and random non-target letters in the remaining squares. The location of the letters in the grid was randomized. Participants were instructed to select all of the letters they remembered from the sequence before pressing a “Submit” button. As with the spatial working memory task, the order of participants’ responses was not emphasized nor analyzed. Responses on the letter memory portion of the task were only counted as correct if the participant selected all of the correct letters from the sequence and no incorrect ones.

Note that chance performance differed among the five tasks described above: random guessing would be expected to yield 50% correct in the towers and mental rotation tasks, 20% correct in the paper folding task, and close to nothing correct in the working memory tasks (0.024%). These differences in chance level did not hinder our ability to compare across tasks, but they did yield numerically different accuracy ranges for the different tasks (see Fig. 2e). Importantly, accuracy on all of the tasks spanned the range between chance and perfect performance and did not cluster near floor or ceiling, providing the desired dynamic range in performance for leveraging individual differences to assess the relationships among the tasks.

### Analyses

Randomized split-half analyses were performed for all of the tasks to characterize the reliability of the measures within our sample. To do so, the trials for each task were randomly split into halves, maintaining an equal number of each trial type in each half of the data. We then computed accuracy on each independent half of the data and computed the correlation of the split halves across participants. All *r* values in the study reflect Pearson correlations.

We performed all significance testing with non-parametric permutation analyses (Ernst, 2004). To do so, we computed the true effect size (for example, the *r* value for a split-half correlation) and then generated a permuted null distribution that captured the range of effect sizes expected by chance. To generate the permuted null distribution, on each of 10,000 iterations, we randomly shuffled the labels of the data (e.g., shuffled the correspondence between a participant’s data in split 1 and split 2 of the data) and recomputed the correlation. The 10,000 resulting *r* values characterize the range of correlations expected by chance, since the true correspondence between halves was destroyed by the shuffling procedure. We then computed the two-tailed significance as the proportion of the permuted null distribution that was larger in absolute value than the true measure effect.

To evaluate whether performance on the spatial and working memory measures could fully account for individual differences in performance on the towers task, we tested for reliable remaining variance in the towers task performance after regressing out the spatial and working memory measures. To do so, we performed a split-half analysis in which we first split the trials from the towers task as described above. We then fit separate multiple regression models to *each* split half of the data. There were two regressors in each model: a combined spatial reasoning variable (the average of performance on the two spatial tasks) and a combined working memory variable (average performance from the two working memory tasks). We used these combined measures after failing to find evidence that the two spatial measures captured distinct variance in towers task performance, and likewise with the two working memory measures (see Results). We obtained the residuals from each regression model and computed the correlation between the residuals from the two split halves of the data. Significant correlation in the residuals would indicate the presence of systematic variation in the Unstable Towers task after accounting for participants’ spatial reasoning and working memory abilities.

## Results

We first set out to construct a task that could reliably measure individual differences in physical prediction abilities. We used the well-established Unstable Towers task as a starting point (Battaglia et al., 2013; Fischer et al., 2016; Hamrick et al., 2016), and created a new set of unstable tower stimuli using the Blender software (http://www.blender.org; see “Methods”). We began with a larger set of candidate stimuli and conducted online piloting to identify a set of towers for which performance was distributed approximately uniformly across the range between chance and perfect performance. We took this approach in order to include towers that spanned the full range of difficulty and would be maximally sensitive to individual differences in performance. Our final stimulus set contained 48 tower stimuli (see Fig. 1a for examples). Data reported for this study were collected from a new, independent set of participants from the piloting process.

The central aim of this study was to test the degree to which physical prediction abilities covary with measures of spatial cognition and working memory across individuals. To this end, we tested 100 online participants on the Unstable Towers task along with four other tasks depicted in Fig. 2: the Paper Folding Test (Ekstrom et al., 1976) and Mental Rotation Test (Peters et al., 1995; Shepard & Metzler, 1971; Vandenberg & Kuse, 1978) as measures of spatial manipulation abilities, and spatial working memory and verbal working memory tasks based on commonly used complex span tasks (Blacker, Negoita, Ewen, & Courtney, 2017; Chein & Morrison, 2010). Each participant completed the five tasks in a random order and was required to work on only one task at a time (see “Methods”).

We first characterized the reliability of the Unstable Towers task using a split-half analysis. We split the stimuli into two sets and computed participants’ performance on each independent half of the stimuli. There was significant positive correlation in participants’ performance on the two halves of the stimulus set (Fig. 1b; *r* (98) = .50, *p* < .001) - a participant’s performance on one half of the stimuli served as a good predictor of performance on the second, independent half. Note that because the stimulus set was divided in half to compute this correlation, it represents a lower bound on the reliability of the towers measure when computing correlation with the remaining four tasks - using the full dataset will yield a less noisy measure of participants’ physical prediction abilities. We also characterized the distribution of performance across individual towers stimuli and measured how reliably easy or difficult a tower was using a split-half analysis (Fig. 1c). Mean accuracy across all towers was 79% correct (SD = 9.2%), and the correlation in performance on the towers in two independent halves of the participant pool was .79 (*r* (46) = .79, *p* < .001), indicating that the difficulty of each stimulus was reliable across independent groups of participants.

**Fig. 3:**
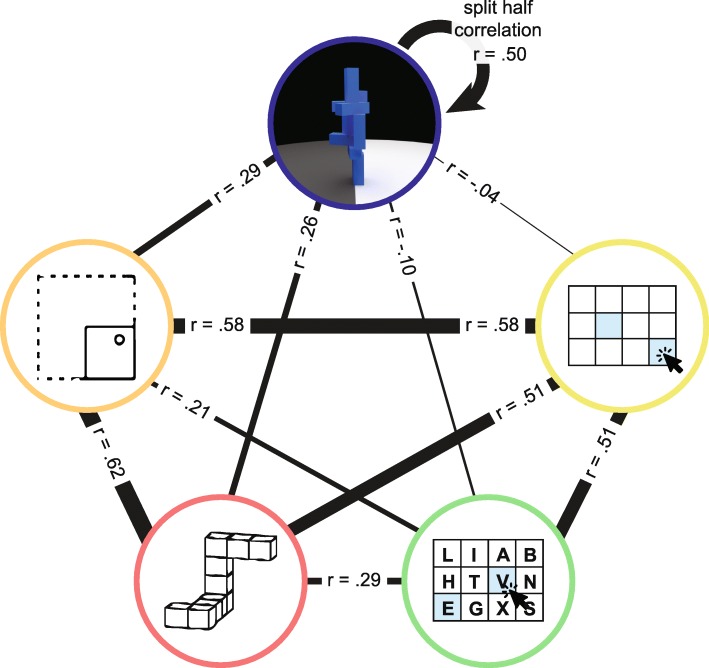
Relationships in participants’ performance among all tasks. Each color-coded circle represents one task. From the top counter-clockwise: Unstable Towers Task, Paper Folding Test, Mental Rotation Test, verbal working memory task, spatial working memory task. The width of each connecting line is scaled to represent the strength of the correlation between individual differences on each pair of tasks. All *r* values are from Pearson correlation tests in 100 participants. All correlations were statistically significant at an alpha level of .05 except for the comparisons of the Unstable Towers task to the verbal and spatial working memory tasks, for which the *p* values were both greater than 0.3. While performance on the Unstable Towers task was significantly correlated with performance on each of the spatial cognition measures, the split-half reliability of the towers task was greater than its correlation with either spatial measure

We then turned our attention to evaluating the relationship between performance on the Unstable Towers task and the remaining four spatial and working memory tasks. Figure 2e shows split-half reliability for each of the spatial and working memory measures. There was strong, significant correlation across independent halves of all of the test items (all *p* values <.001), indicating good reliability of the measures. Figure 3 shows the correlations between individual differences in performance among all five tasks in the study, using the full data set from each task. We identified significant positive correlation between performance on the Unstable Towers task and the Paper Folding Test (*r* (98) = 0.29, *p* = .0021) and Mental Rotations Test (*r* (98) = 0.26, *p* = .0098). On the other hand, to our surprise, we found no significant correlation between performance on the towers task and either measure of working memory (spatial working memory: *r* (98) = − 0.038, *p* > .5; verbal working memory: *r* (98) = − 0.10, *p* = .32). The lack of correlation was not due to low reliability of the working memory tests as indicated above, and the working memory tests correlated significantly with each other (*r* (98) = 0.62, *p* < .001) and with the measures of spatial cognition (the largest *p* value was .040). We explore possible reasons for the lack of correlation between the towers task and the working memory tasks in “Discussion”.

The data above indicate that performance on each of the spatial tasks served as a significant predictor of physical inference abilities in the towers task, and the two spatial measures were correlated with the towers task to roughly the same degree. We next evaluated whether the two spatial tasks captured shared or partially distinct variance in towers performance. We conducted a model comparison procedure in which we compared the fit of a model with only the Paper Folding Task as a predictor with that of a second model including both the Paper Folding Task and the Mental Rotation Task as distinct predictors. The second model captured more variance but also included an additional free parameter, and the model comparison tested which model was more likely the correct one using the Akaike information criterion (AIC) (Akaike, 1998). The model comparison favored the simpler one-parameter model (AIC_model1_ = − 481.07, AIC_model2_ = − 480.03; model 1 was 1.7 times more likely to be the correct model according to the information ratio), and there was no significant additional variance captured by adding the second parameter to the model (*F* (98,97) = 1.10, *p* = .30). We conducted the same test for the two working memory measures and found that a model predicting towers task performance from the verbal working memory alone (AIC = − 473.03) was 2.9 times more likely to be the correct model than one that included both working memory measures as separate parameters (AIC = − 470.87), and no significant additional variance was explained by adding the second parameter (*F* (98,97) = 0.01, *p* = .91). Thus, moving forward, we used combined measures (computed by averaging across tasks) to capture spatial abilities in a single predictor and working memory performance in another single predictor. As expected, the combined measure of spatial abilities significantly predicted performance on the towers task (*r* (98) = 0.31, *p* = .0019) while the working memory measure did not (*r* (98) = − 0.075, *p* = .45).

**Fig. 4:**
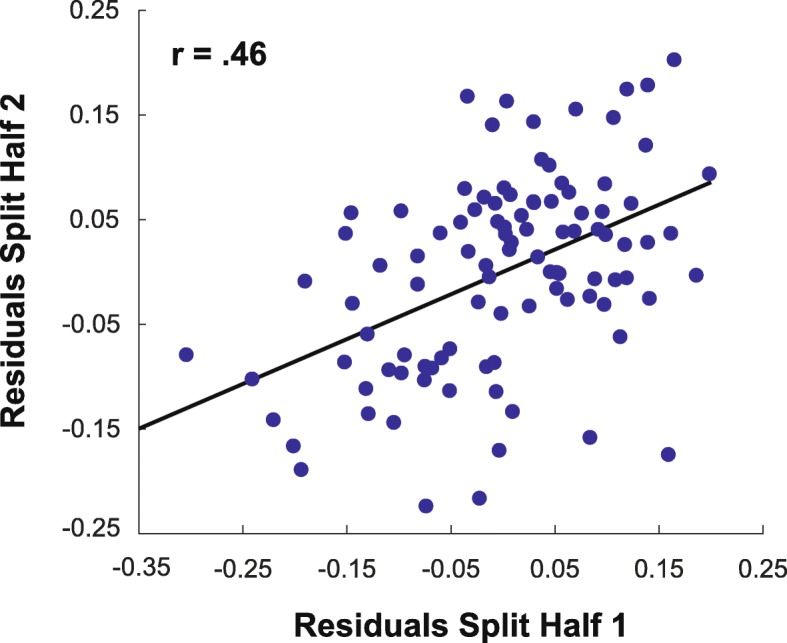
Reliable variance in the Unstable Towers task after regressing out the contribution of spatial abilities and working memory. We ran a linear regression to find the degree to which a combination of the spatial and working memory measures could account for individual differences in performance on the towers task (see “Methods”). We then found the residual (unexplained) variance in the towers task and plotted independent split halves of the data (each data point represents one participant). We identified robust positive correlation in this split-half analysis (*r* (98) = 0.48, *p* < .001), indicating that reliable individual differences in physical prediction cannot be fully accounted for by spatial abilities or working memory capacity

In our final analysis, we sought to test whether performance on the spatial and working memory measures could fully account for individual differences in performance on the towers task. To do so, we separately fit linear regression models to independent split halves of the towers data with the combined spatial and working memory measures as regressors in each model (see “Methods”). We then took the residuals from these models (reflecting unexplained variance in each split half) and asked whether there was reliable variation across individuals (Fig. 4). We identified strong and significant split-half correlation in the residuals (*r* (98) = 0.46, *p* < .001), demonstrating that there were systematic, reliable variations in physical prediction performance that could not be explained by spatial abilities or working memory. These results demonstrate the separability of performance on the Unstable Towers task from measures of both spatial cognition and working memory. While the spatial measures were significant predictors of physical inference abilities, the facets of intuitive physics captured by the towers task are not simply an extension of spatial manipulation abilities.

## Discussion

This study assessed the separability of physical prediction abilities from spatial reasoning and working memory. After developing a set of Unstable Towers stimuli that could reliably capture individual differences in physical prediction performance, we tested the relationship between this intuitive physics task and four other well-established measures of spatial reasoning and working memory. While our results show that spatial abilities are a significant predictor of performance on the towers task, we found reliable individual differences in physical inference abilities that could not be accounted for by either spatial abilities or working memory. These results point to the separability of intuitive physics from spatial cognition - our physical intuitions are not merely an extension of our spatial thinking.

At the outset of this study, there was good reason to suspect that intuitive physics and spatial cognition would by closely related. During development, for example, the emergence of spatial thinking and physical concepts is intertwined. The looking behaviors of infants at 7 months old indicate that they are able to represent how the geometric properties of an object, such as the length and width, influence how it will physically interact with other objects in a scene (Baillargeon, 1987). Further, infants of the same age can recognize how the physical properties of an object (i.e. soft/malleable vs hard) relate to spatial changes when interacting with other objects. In adults, a study on individual differences in spatial abilities and mechanical reasoning found that participants high in spatial ability made significantly fewer errors on predicting how a pulley system would operate (Hegarty & Sims, 1994), and recent work on people with Williams syndrome who display characteristic spatial processing deficits showed that they also have marked difficulty in performing the Unstable Towers task (Kamps et al., 2017). Additionally, a recent study that identified the brain regions engaged during physical inference (Fischer et al., 2016) revealed a set of regions that is highly similar to those engaged during spatial manipulation tasks (Richter et al., 2000; Vingerhoets et al., 2002). Thus, the positive relationship between intuitive physics and spatial cognition was expected, but the degree to which the two were separable at all had not been established.

We also expected that performance on the working memory tasks would be a good predictor of physical prediction abilities. Tracking the numerous objects involved in physical interactions must tax working memory, and physical prediction and working memory tasks engage highly overlapping sets of brain regions (Fischer et al., 2016). To our surprise, we found no relationship between the working memory measures and physical prediction performance in our current tasks, despite the fact that each measure showed strong and significant internal reliability. Why were intuitive physics and working memory unrelated in our data? One possibility is that the Unstable Towers task may be an example of a kind of prediction that we perform without explicitly tracking individual objects within the scene. We may perform the prediction in a more holistic fashion, assessing ensemble statistics (Alvarez, 2011; Haberman & Whitney, 2012) that are predictive of the final outcome state. The number of blocks in the towers we presented here (11–21 blocks) is far greater than the number of items that we can track individually, yet observers still generally perform well at the task. Participants may also only attend to a critical subset of the blocks that are key in determining the behavior of the tower as a whole. It may be the case, then, that the Unstable Towers task places low demands on working memory, accounting for the lack of a relationship in our data. Importantly, even if this is the case, it still points to the separability of intuitive physics from working memory - we can solve physical prediction problems without placing a high demand on working memory. In future work, it will be important to extend this present approach to other intuitive physics tasks that are more likely to tax working memory.

What is the source of variability in physical prediction abilities across individuals? At least two (non-mutually exclusive) possibilities merit consideration. First, there may be differences across individuals in the precision and capacity of the mental physics engine itself. To form their predictions, people must represent physical information about the objects and surfaces in the environment (e.g., friction, mass, and deformability) and they must have stored knowledge about physical constants and principles such as the gravitational constant (Indovina et al., 2005; Zago & Lacquaniti, 2005). Even if everyone employs roughly the same algorithms to carry out their physical inferences, individual differences in the precision with which the above properties are represented would manifest as differences in prediction accuracy. The precision and scope of the inference processes themselves could also differ across individuals. Imagining for a moment that people do use some form of mental simulation to generate their physical predictions, there may be individual differences in the speed and temporal resolution with which simulations can be carried out, the spatial precision of simulations, or even the number of simulations that can be carried out before reaching a decision, all of which could lead to variations in error rates across individuals. A second possibility is that people differ qualitatively with regard to their strategies for forming predictions. In the Unstable Towers task used here, individuals may differ in the portions of the towers they focus on (e.g., blocks at the top or the bottom of each tower) or any shortcuts they use to make their judgements (e.g., relying on the tower’s center of mass as an indicator of how it will fall). This second case may be expected to give rise to more qualitative differences in observers’ predictions than the first, a distinction that may prove useful for future work that aims to adjudicate among these and other possibilities. In any case, day-to-day experience may play an important role. Some individuals rely particularly heavily on their physical inferences in daily life (e.g., athletes or automobile mechanics), and the constant demands on their physical inference abilities may lead to more finely tuned mental physics engines.

As noted in “Introduction”, it is important to acknowledge that the Unstable Towers task used here is just a single task within the broader landscape of intuitive physics, and basic stability judgements like this one may be more automatic and less simulation-based than others where a larger number of variables are at play (Firestone & Scholl, 2017). This task may well reflect a case where we use the mental physics engine to train a fast, bottom-up network to determine how the towers will fall, circumventing the need for full-blown simulation in each new case (Wu, Yildirim, Lim, Freeman, & Tenenbaum, 2015). Thus, this study is a starting point in a broader endeavor to examine the relationship between a range of intuitive physics tasks and a range of cognitive abilities that are likely related (e.g., spatial and featural attention, temporal prediction, and many others). Still, the present findings are sufficient to establish that the aspects of intuitive physics captured by this task cannot be fully accounted for by spatial abilities or working memory.

Amidst our findings here on what intuitive physics is *not* (i.e., simply a recruitment of spatial cognition and/or working memory), it is worth putting forward a theoretical description of what we believe the mental physics engine *is*. In our conception, the mental physics engine comprises a collection of cognitive processes that apply a simplified set of physical laws to a multimodal representation of the world. The products of these processes are at least fourfold: (1) a physical scene description that includes information about how objects rest on or support each other, whether objects and surfaces that are in contact are attached or free moving, and what the latent physical properties (e.g., mass, hardness, and friction) are for the constituents of the scene; (2) probabilistic predictions of how the physical dynamics in a scene will play out over time. No matter what algorithms underlie physical prediction (e.g., mental simulation, rule-based reasoning, or some combination thereof), the outcomes are likely probabilistic - e.g., we see that a rolling pen will most likely fall from the table but may stop just short; (3) inferences about what past physical conditions gave rise to the current ones. A key function of the mental physics engine is to infer possible causes from observed effects; for example, that a trail on a sandy beach was formed by a rolling ball (Gerstenberg, Siegel, & Tenenbaum, 2018) (for related work on shape perception, see Chen & Scholl, 2016; Leyton, 1989; Spröte, Schmidt, & Fleming, 2016); and (4) estimates of how our own actions and the actions of others will influence the world. Perhaps the most crucial function of the mental physics engine is to allow us to plan and select among possible actions by evaluating the outcome that each action would likely lead to. These four kinds of information computed by the mental physics engine do not necessarily arise from distinct processes - rather, they are likely computed jointly and in a mutually informative manner. To achieve such a general and flexible set of inferences that are useful across the vast array of physical scenarios that we encounter in daily life, a physical simulation engine is an attractive model (Battaglia et al., 2013; Ullman et al., 2017). The aforementioned computations would be achieved by storing physical variables about the objects and surfaces in a scene and stepping forward in time through successive states of the world, applying simplified physical laws to determine the outcomes of events such as collisions. It remains possible that an entirely different mechanism is at play, e.g. the application of large collection of rules and heuristics (Davis & Marcus, 2016; Ludwin-Peery, Bramley, Davis, & Gureckis, 2019). However, given the flexibility of our physical reasoning in the face of the novel scenarios we encounter on a daily basis, we favor an account in which we rely primarily on mental simulation but supplement our simulations with heuristics in cases where simulation is intractable or unnecessary (e.g., evaluating whether water will escape a sealed container), and in cases where specific forms of physical prediction are overlearned (e.g., a player catching a baseball). In any case, the mental architecture underlying intuitive physics should be malleable, with the capacity to be elaborated and fine-tuned through experience, both perceptual and motor. Indeed, there is evidence that motor experience can enhance the understanding of physical concepts, both during development (Rakison & Krogh, 2012) and in adulthood (Kontra, Lyons, Fischer, & Beilock, 2015).

Our findings here show that an individual’s intuitive physics abilities (as measured by the Unstable Towers task) cannot be fully explained by his or her spatial skills alone, but does that necessarily mean that the two systems are distinct in the mind? Since most physical predictions involve some form of spatial manipulation in order to understand how objects will move and reorient over time, one might argue that spatial thinking is subsumed by the mind’s physics engine, and spatial tasks constructed by experimenters simply tap into one component of a more general simulation engine. Our data are consistent with this possibility - the separability between intuitive physics and spatial cognition that we identified may arise from other facets of the mental physics engine that are untapped by purely spatial tasks (e.g., the incorporation of physical properties such as gravity, mass, and friction and application of physical laws handing behaviors such as colliding, sliding, and falling). The distinction is a subtle but important one for future work to resolve - is spatial cognition a “pipe” that feeds into the mental physics engine, or part of the physics engine itself? Still, this lingering question does not undermine our central claim: intuitive physics is more than just spatial cognition, whether they are carried out by shared or distinct mental machinery. It is worth noting that some spatial computations, e.g. those that give rise to the perceived positions and sizes of objects, emerge early in the cortical visual processing stream (Fischer, Spotswood, & Whitney, 2011; Maus, Fischer, & Whitney, 2013; Murray, Boyaci, & Kersten, 2006) in regions that are not specifically engaged during physical prediction (Fischer et al., 2016). Still, as mentioned above, spatial tasks like those employed here do engage highly similar sets of brain regions to those involved in intuitive physics (Richter et al., 2000; Vingerhoets et al., 2002), so the question of distinct mental machinery remains unresolved from the standpoint of brain imaging research as well.

In summary, we found that individual observers display reliable variability in their physical prediction abilities that cannot be accounted for by their spatial abilities or working memory capacity. We developed a new set of physical prediction stimuli to characterize such variability, and demonstrated that it has strong internal reliability to capture individual differences. Our findings point to the possibility that we may possess some specialized, domain-specific mental resources that support our physical intuitions and allow us to interact fluidly with our everyday environments.

## Data Availability

The data and full stimulus set from this study are available at http://www.dynamicperceptionlab.com/tasks.
